# Hierarchically Annular Mesoporous Carbon Derived from Phenolic Resin for Efficient Removal of Antibiotics in Wastewater

**DOI:** 10.3390/molecules27196735

**Published:** 2022-10-09

**Authors:** Xuexia Lin, Mengxing Su, Feixiang Fang, Jiafu Hong, Yumeng Zhang, Shu-Feng Zhou

**Affiliations:** 1 Department of Chemical Engineering & Pharmaceutical Engineering, College of Chemical Engineering, Huaqiao University, Xiamen 361021, China; 2 State Key Laboratory for Marine Corrosion and Protection, Luoyang Ship Material Research Institute, Xiamen 361101, China

**Keywords:** mesoporous carbon, adsorption, penicillin, streptomycin, tetracycline hydrochloride, wastewater

## Abstract

Antibiotics have become a new type of environmental pollutant due to their extensive use. High-performance adsorbents are of paramount significance for a cost-effective and environmentally friendly strategy to remove antibiotics from water environments. Herein, we report a novel annular mesoporous carbon (MCN), prepared by phenolic resin and triblock copolymer F127, as a high-performance adsorbent to remove penicillin, streptomycin, and tetracycline hydrochloride from wastewater. The MCNs have high purity, rich annular mesoporosity, a high surface area (605.53 m^2^/g), and large pore volume (0.58 cm^3^/g), improving the adsorption capacity and facilitating the efficient removal of penicillin, streptomycin, and tetracycline hydrochloride from water. In the application of MCNs to treat these three kinds of residual antibiotics, the adsorption amounts of tetracycline hydrochloride were higher than penicillin and streptomycin, and the adsorption capacity was up to 880.6 mg/g. Moreover, high removal efficiency (99.6%) and excellent recyclability were achieved. The results demonstrate that MCN adsorbents have significant potential in the treatment of water contaminated with antibiotics.

## 1. Introduction

Antibiotics are widely used for controlling diseases in humans, animals, and plants because of their ability to treat diseases caused by infections [1,2]. However, it has been reported that large amounts of antibiotics not completely absorbed or metabolized are excreted into surface water, groundwater, and even drinking water [3,4]. The residual antibiotics not only exacerbate the shortage of water resources, but also pose potentially serious threats to human health and ecosystems [5]. Therefore, it is essential to remove antibiotic residues from various kinds of water environments.

Over the past decade, biological treatments and advanced oxidation technologies were developed to remove residual antibiotics from water environments. The biological treatment techniques commonly require the combination of various kinds of microorganisms to remove residual antibiotics and improve their poor biodegradability [6,7]. Although advanced oxidation processes are quick and effective, they have a considerable demand for energy and chemicals [8,9]. Moreover, ion exchange and liquid membrane separation often use chemical regents that could cause secondary pollution in the natural environment [10,11]. Given these limitations, adsorption is still the preferred choice for the removal of residual antibiotics from wastewater due to its advantages of simplicity, low cost, and high efficiency, and because it does not produce intermediate products.

Various adsorption materials have been studied, including activated carbon [12], carbon nanotubes [13], biochar [14], and others [15]. Although biochar as a cheap carbon adsorbent has become a research focus, it remains crucial to develop high-performance carbon adsorbents with simple preparation and high purity for improving removal efficiency by refining their porous structure, carbon skeleton, and so on. Mesoporous carbon nanoparticles (MCNs) can impact the MCN–contaminant interaction, leading enhanced adsorption efficiency and removal efficiency given their fine pore structure, open structural framework, high surface areas, large pore volume, mechanical stability, and biocompatibility [16,17,18]. Recently, Fang and coworkers synthesized highly ordered body-centered cubic (*Im^3^m*) MCNs with line-type channels by a novel low-concentration hydrothermal route [19]. Liu et al. reported facile annular small mesoporous hydrothermal nanospheres with tunable sizes of 30 to 80 nm, ordered channels, and abundant functional glycosylation groups [20]. Yang and co-workers fabricated mesoporous carbon spheres using amphiphilic Pluronic F127 as the surfactant, 1,3,5-trimethyl benzene as the pore swelling and interface-adjusting agent, and dopamine as the carbon source [21]. The mesoporous carbon spheres were successfully synthesized with tunable pore sizes and versatile architectures, such as vesicles, walnut shapes, spheres with dendritic-like 3D radially aligned mesochannels, and isolated spherical mesopores. More importantly, the particle sizes of MCNs are smaller than 200 nm, which can provide short pathways for mass transport and minimize the viscous effects. However, using MCNs as adsorbents for the adsorption of bulky molecules has not yet been fully realized.

In this work, we synthesized MCNs for the removal of residual antibiotics from wastewater based on the application of F127 and phenolic resin to synthesize MCNs through the self-assembling approach and the hydrothermal method. The structure and properties of the synthetic MCNs were characterized by SEM, TEM, Raman spectroscopy, FTIR spectroscopy, XRD patterns, and SAXS. In addition, we systematically investigated adsorption parameters including absorption time, absorption capacity, removal efficiency, and recycle times.

## 2. Results and Discussion

### 2.1. Characterization of MCNs

**Figure 1:**
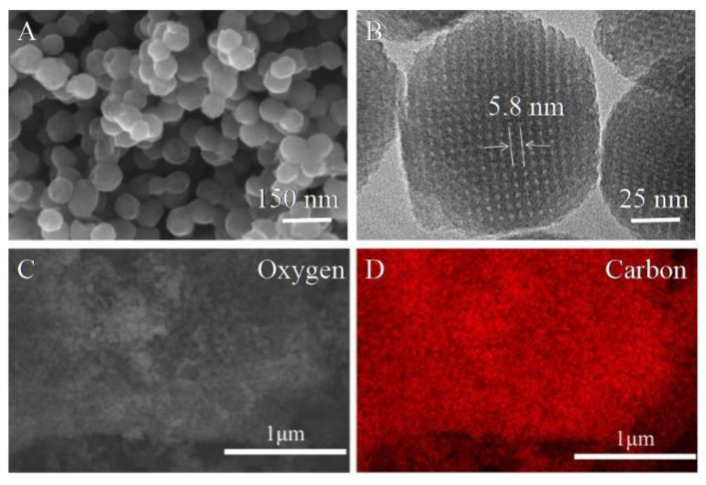
Structural characterization of annular surface MCNs. (**A**) SEM, (**B**) TEM, (**C**) element analysis of MCNs by EDS, and (**D**) carbon element analysis of MCNs.

The mesoporous carbons were synthesized via the surfactant self-assembling approach by using preformed phenolic resins as carbon sources and triblock copolymer F127 with a high EO/PO ratio as a structure-directing agent. The morphologies of the MCNs were investigated by SEM and TEM. As shown in Figure 1A, MCNs have regular morphology, particular annular pores on the particle surface, and obvious pores. The particle sizes of MCNs are about 50 nm. As can be observed from Figure 1B, TEM images of MCNs show annular mesoporous channels arranged orderly. MCNs have both line-type and annular channels. A single channel of the annular MCNs was measured as approximately 5.8 nm, and the unit is F127 rod. In addition, the results of EDS analysis in Figure 1C,D exhibit that MCNs consist of 90.48% carbon and 2.6% oxygen elements with little mineral constituents of Si and Cu, indicating their high purity. This can be attributed to their preparation mechanism. Phenolic resin and triblock copolymer F127 as the precursors merely contained carbon and oxygen, which is different from biochar that often retains metallic inorganic elements. The higher purity of MCNs indicate that they have more adsorption sites for interacting with residual antibiotics. Thus, MCNs could potentially have efficient adsorption properties for pollutants’ removal from water. 

**Figure 2:**
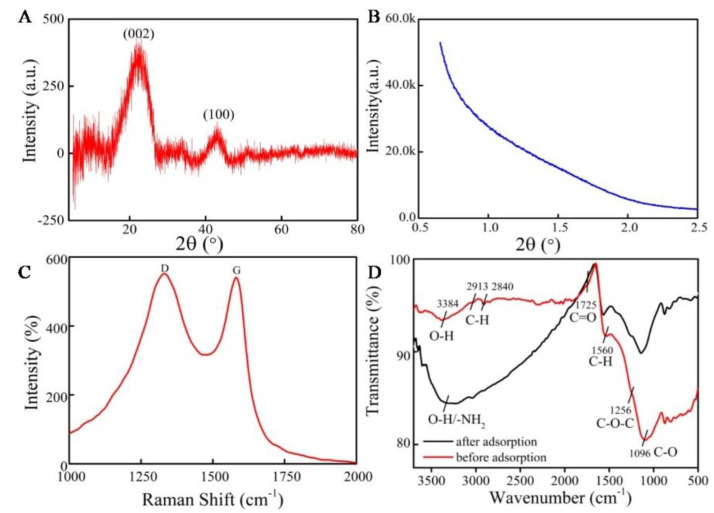
Characterization of MCNs by (**A**) XRD patterns, (**B**) SAXS patterns, (**C**) Raman spectra, and (**D**) FTIR spectra.

The great peak strength of MCNs illustrates that functional groups may exist on its surface. The structure of the as-synthesized MCNs was investigated by XRD and SAXS patterns. Figure 2A shows characteristic peaks at 2θ = 24.96° and 45°, which correspond to (002) and (100) planes, respectively. The results of SAXS show that MCNs have uniform pores. These results demonstrate that MCN-3 has a smaller size and a more disordered structure, as shown in Figure 2B. The Raman spectra are shown in Figure 2C. It can be clearly observed that two characteristic bands were observed at about 1345 cm^−1^ (D-band) and 1590 cm^−1^ (G-band), where the D band corresponds to the sp^3^ carbon atoms and the G band corresponds to the sp^2^ carbon atoms in the nanographite structure. Raman spectra of MCNs show that the intensity ratio of the G peak and the D peak (ID/IG) is about 1, indicating that the content of the sp^2^ bonding is similar to that of the sp^3^ bonding [22]. Additionally, the FTIR spectra of MCNs are shown in Figure 2D. The characteristic peak at 3379 cm^−1^ was assigned to O-H stretching vibration, mainly related to the carboxyl and phenolic hydroxyl groups on MCNs, and peaks at around 2913 and 2840 cm^−1^ correspond to C-H stretching vibration, related to CH_2_ and CH_3_. The peak at about 1560 cm^−1^ would be the C=O vibration in aromatic groups. The peak at around 1256 cm^−1^ is the C-O-C asymmetric telescopic vibration. The peak at 1096 cm^−1^ is the C-O stretching of phenol. The peaks at 881–690 cm^−1^ are the C-H bending vibrations of aromatic groups. Moreover, it can be observed that after adsorption, the peak at 3379 cm^−1^ of O-H stretching vibration and N-H vibration becomes larger, and the peak of acyl group vibration (C=O) becomes smaller. These changes indicate that the functional groups of MCNs are altered, and the larger peak at 3379 cm^−1^ indicates that antibiotics would be absorbed because the antibiotics are rich in hydroxyl groups and amino groups. 

**Figure 3:**
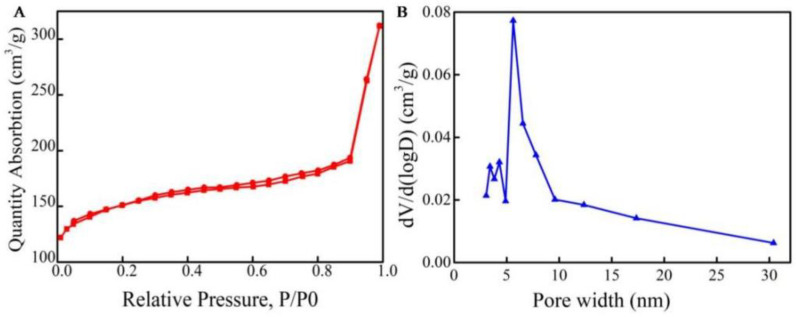
Brunauer–Emmett–Teller (BET) surface area and pore size distribution curves. (**A**) N_2_ adsorption–desorption isotherms of MCNs. (**B**) Pore size distribution of MCNs.

The BET of MCNs exhibits representative type IV curves with a hysteresis loop, revealing the presence of both micropores and mesopores [23]. On the low-pressure side (0.0–0.1), a strong force was shown between MCNs and nitrogen. It has obvious capillary condensation steps at P/P_0_ > 0.4, suggesting a rich mesoporous structure [24]. In Figure 3, the corresponding pore size distributions, calculated based on the Barrett-Joyner-Halenda (BJH) model, indicate that the pore size distribution of the mesoporous carbon materials was mainly concentrated at around 5–7 nm. It is verified that the MCNs’ pore structures are the same and their pore diameters are consistent with the TEM images. In addition, the specific surface area of MCNs was calculated as 358.8 m^2^/g. The relevant pore volume and pore size are about 0.31 cm^3^/g and 5.67 nm, respectively. The large specific surface areas and reasonable porous structures would provide more active sites to interact with residual antibiotics and facilitate the adsorption process.

### 2.2. Adsorption of Antibiotics on MCNs 

#### 2.2.1. Adsorption Isotherm Study 

The isotherm adsorption measurements were conducted by adding 100 mg of MCNs in 100 mL of the three antibiotics solutions. The adsorption behavior isotherm data of three antibiotics were evaluated by the Langmuir (Equation (1)) and Freundlich (Equation (2)) isotherms.

(1) $$Q_{e}=\frac{k_{L}Q_{m}C_{e}}{1+k_{L}C_{e}}\;\;\;\;$$

(2) $$\;\;\;\;\;\;\;Q_{e}=k_{f}C_{e}^{\frac{1}{n}}\;\;\;$$

where *Q_m_* (mg/g) is the saturated adsorption capacity. *k_L_* (L/mg) and *k_F_* ((mg/g^−1^)/(mg/L)^n^) represent the Langmuir equilibrium constant and Freundlich constant, respectively. n is the Freundlich intensity parameter that represents the magnitude of the adsorption driving force or surface heterogeneity.

**Figure 4:**
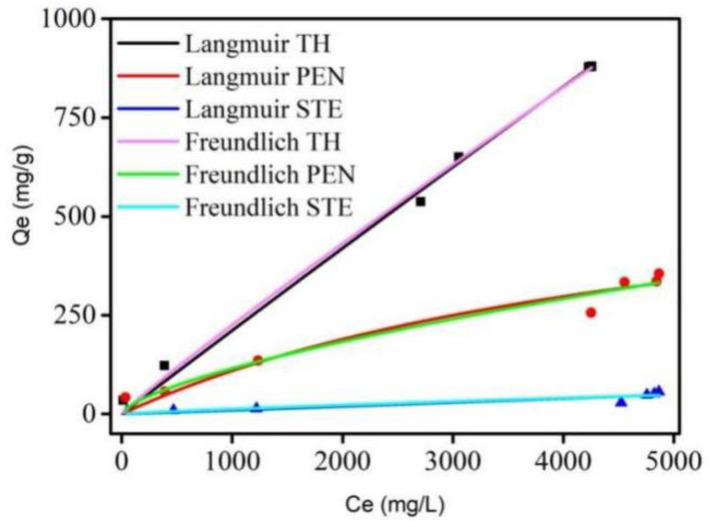
The Langmuir isotherms and Freundlich isotherms for streptomycin, penicillin, and tetracycline hydrochloride adsorption on MCNs at 37 °C.

The adsorption behaviors of MCNs were evaluated by the Langmuir and Freundlich isotherms in Figure 4. Adsorption parameters of these two models are listed in Table 1. The results of R^2^ values of the Langmuir model are similar to those of the Freundlich model, and there is little difference. These results may be related to the monolayer and poly-molecular layer adsorption of the three kinds of antibiotics on MCNs. High values of *Q_m_* (693 mg/g~31,497 mg/g) from the Langmuir model indicate a high specific surface area. These values also prove that MCNs with high purity and high porosity have effective adsorption performance, especially adsorption capacity. The values of 1/n from the Freundlich model ranged from 0.6 to 1.0, indicating the high affinity between antibiotics and MCNs and suggesting that the adsorption is favorable. These data imply that MCNs have promising potential for antibiotic removal from wastewater.

#### 2.2.2. Adsorption Kinetics 

**Figure 5:**
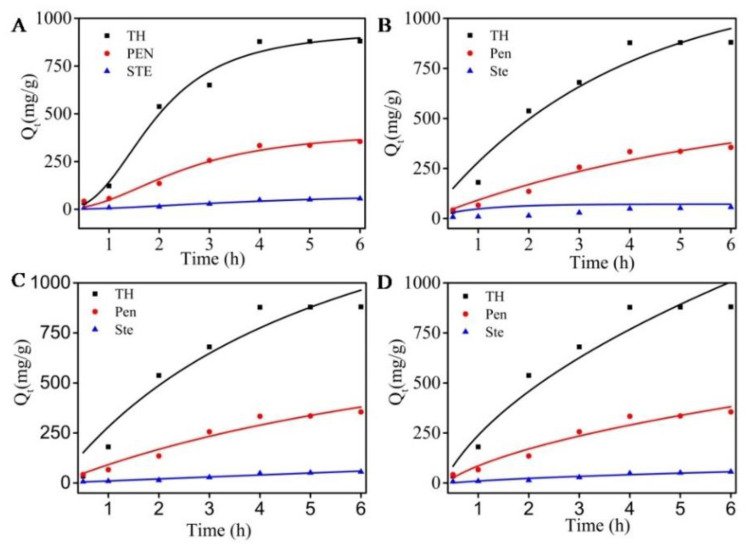
The adsorption kinetics of three kinds of antibiotics on MCNs. (**A**) Adsorption effect of MCNs on antibiotics. (**B**) Pseudo-first-order kinetic curve based on Equation (6). (**C**) Pseudo-second-order kinetic curve based on Equation (8). (**D**) Intraparticle diffusion curve based on Equation (9).

**Figure 6:**
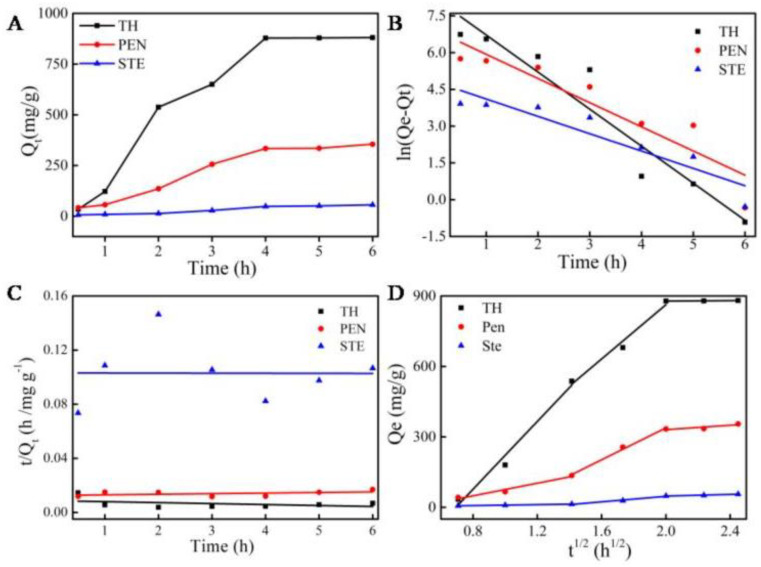
The adsorption kinetics of three kinds of antibiotics on MCNs with linearity. (**A**) Adsorption effect of MCNs on antibiotics. (**B**) Pseudo-first-order kinetic curve. (**C**) Pseudo-second-order kinetic curve. (**D**) Intraparticle diffusion curve.

We analyzed the kinetic behavior of streptomycin, penicillin, and tetracycline hydrochloride adsorption on MCNs to understand the relationship between the adsorbed amount and time. As shown in Figure 5A and Figure 6A, by using an initial concentration of 20 mg/L of streptomycin, penicillin, and tetracycline hydrochloride, MCNs showed the fastest adsorption rate in the first 2 h because of the presence of active sites. As time increased from 1 h to 4 h, the adsorption rate increased, and then achieved equilibrium after 4 h. 

The adsorption capacities of streptomycin, penicillin, and tetracycline hydrochloride are 56.2 mg/g, 355.3 mg/g, and 880.6 mg/g, respectively. We investigated adsorption kinetics by the pseudo-first-order kinetic (PFO, Equations (3) and (4)), pseudo-second-order kinetic (PSO, Equations (5) and (6)), and intra-particle diffusion (IPD, Equations (7) and (8)) models.

(3) $$Q_{t}=Q_{e}\left(1-e^{-k_{1}t}\right)$$

(4) $$ln\left(Q_{e}-Q_{t}\right)=lnQ_{e}-k_{1}t$$

(5) $$Q_{t}=\frac{k_{2}Q_{e}^{2}t}{1+k_{2}Q_{e}t}$$

(6) $$\frac{t}{Q_{t}}=\frac{t}{k_{2}Q_{e}^{2}}+\frac{1}{Q_{e}}t$$

(7) $$Q_{t}=k_{3}t^{0.5}+c$$

(8) $$Q_{t}=k_{3}\sqrt{t}+c$$

where *Q_e_* and *Q_t_* represent the adsorption capacity at equilibrium time and any time, respectively. *k_1_*, *k_2_*, and *k_3_* are the PFO, PSO, and IPD kinetic constant, respectively. t is the absorption time and c is a constant related to the thickness of the boundary layer.

The correlation values of PFO (R^2^ > 0.830) are greater than the PSO kinetic model (R^2^ < 0.04) in Table 2, which demonstrates that the adsorption of the three antibiotics on MCNs is mainly controlled by the PFO kinetic model. The high PFO kinetic constant values (*k_1_*) and high *Q_e_* in Table 2 indicate that MCNs have a fast adsorption rate and high adsorption capacity for tetracycline hydrochloride, penicillin, and streptomycin. Moreover, as shown in Figure 5 and Figure 6, the rate of adsorption and the adsorption capacity for the three antibiotics follow the order of tetracycline hydrochloride > penicillin > streptomycin. 

According to the IPD model, all data points can be divided into three linear segments, shown in Figure 5D, Figure 6D and Table 3. All three segments do not cross the origin, which indicates that the internal diffusion is dominant in the rate-limiting steps but the boundary layer is also involved. The first slope corresponds to the mass transport of antibiotics to the surface of MCNs. The second slope shows that the antibiotics migrated into the mesopores of MCNs. The third slope indicates the antibiotics’ diffusion in the micropores of MCNs. In the final stage, the high Ci intercept value denotes the increased mass transfer resistance. Compared with the intra-particle diffusion rate constant k in Table 3, we can infer that the adsorption speed of tetracycline is the fastest, that of streptomycin is the second fastest, and that of penicillin is the slowest. The higher *k_1_* of tetracycline hydrochloride indicates that the absorption of tetracycline hydrochloride has a higher adsorption rate and more adsorption sites. *k_2_* and *k_3_* indicate the adsorption rate of mesopores and micropores in Figure 6D and in Table 3. For tetracycline hydrochloride, the values of *k_1_* and *k_2_* demonstrate that the main adsorption of streptomycin and penicillin occurs in the mesopores of MCNs, while the main adsorption of tetracycline hydrochloride occurs not only in mesopores but also on the surface of materials. These are the reasons that MCNs’ adsorption capacity and adsorption rate for tetracycline hydrochloride are higher than the other two. Furthermore, the absorption rate could be further improved in future work by the combination of other nanomaterials that can be rapidly adsorbed with targets. In addition, the high *Q_e_* of TH is up to 4268.2 mg/g, which is higher than some current activated carbons such as porous carbon (222.0 mg/g) [25] and biochar (300.82 mg/g) [26].

### 2.3. Removal Efficiency and Reuse of MCNs 

**Figure 7:**
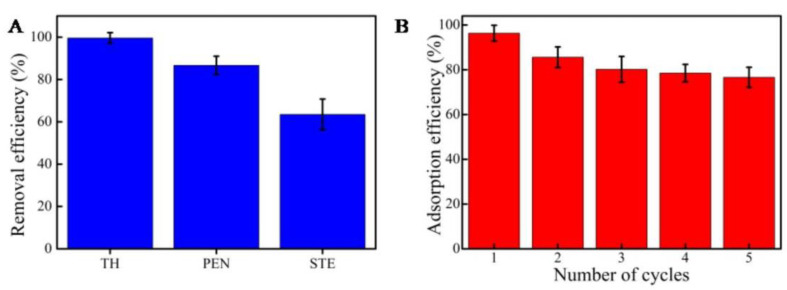
Removal efficiency and reuse of MCNs for adsorption of antibiotics. (**A**) Removal efficiency. (**B**) Number of cycles. All the experiments were performed three times.

In order to estimate the durability of MCNs in practical applications, removal efficiency and reuse experiments were conducted. The removal efficiencies of streptomycin, penicillin, and tetracycline hydrochloride from deionized water by adsorption on MCNs are displayed in Figure 7A. With the initial concentration of 20 mg/L, about 99.6% of tetracycline hydrochloride could be removed in 4 h. The removal efficiency of streptomycin is 86.7%, and that of penicillin is 63.5%. We speculated that the higher removal efficiency of tetracycline hydrochloride is due to high purity of MCNs, which would provide more activated sites of MCNs. Figure 7B shows the adsorption efficiency of MCNs after five adsorption and regeneration cycles. The maximum adsorption capacity of MCNs could reach about 76.6% with stable performance. Finally, the prepared MCNs were applied to remove tetracycline hydrochloride, streptomycin, and penicillin from river and lake water. The removal efficiency rates of streptomycin, penicillin, and tetracycline hydrochloride from the river water sample were about 89.3%, 73.5%, and 52.3%, respectively; in the lake water, the removal efficiency rates were about 92.3%, 75.6%, and 58.3%. The lake water was cleaner than the river water, and there was a certain amount of domestic sewage discharge into the river. Compared with purified water, the removal efficiencies of antibiotics from river water and lake water are relatively low because they contain more inorganic compounds and organic compounds, which would reduce the number of active sites. The high removal efficiency demonstrates that MCN may be a promising adsorbent in environmental applications. Our work demonstrates a possible mechanism for the adsorption of antibiotics on MCNs. The adsorption of antibiotic molecules from an aqueous solution depends not only on the surface characteristics of the MCNs, but it is also greatly affected by the mesoporous structure. 

## 3. Materials and Methods

### 3.1. Reagents and Materials

Pluronic F127 (Mw = 12,600, EO_106_PO_70_EO_106_) was purchased from Sigma-Aldrich Chemicals (Shanghai, China). Phenol, formaldehyde (37%), sodium hydroxide, and alcohol were purchased from Xilong Scientific Co., Ltd. (Shantou, China). Nutritional broth medium, streptomycin, penicillin, and tetracycline hydrochloride were obtained from Beijing Solarbio Science & Technology Corp (Beijing, China). The ultrapure water (18 MΩ·cm^−1^) was obtained from a Milli-Q water purification system (Millipore Corporation, Burlington, MA, USA). All chemicals used in this study were of analytical reagent grade. 

### 3.2. Preparation of MCNs

We used triblock copolymer Pluronic F127 as a structure-directing agent and low-order phenolic resin as a carbon source to prepare a series of mesoporous carbon materials. Firstly, 1.2 g of phenol, 4.2 mL of formaldehyde solution (37%), and 30 mL of 0.1 M sodium hydroxide solution were mixed at 70 °C for 0.5 h. Then, 30 mL of water dissolved with 2 g of Pluronic F127 was added to the solution at 70 °C for 3 h. After that, 100 mL of water was added to dilute the solution. The solution continued to react at 70 °C for 24 h. Then, the solution was cooled down to room temperature. We mixed the solution with water at different volume ratios of 1:4, and these mixed solutions were transferred into a hydrothermal kettle at 130 °C for 24 h. After three cycles of centrifugation (5000 rpm/min, 3 min), washing with water, and drying in dishes at 60 °C for 12 h, the dried samples were carbonized at high temperatures under nitrogen atmosphere. Heating programs were from ambient temperature to 500 °C with a rate of 10 °C/min, keeping this temperature for 2 h to remove the triblock copolymer template, and then from 500 to 700 °C with a rate of 10 °C/min, keeping this temperature for 2 h. The black powders, named MCNs, were collected. 

### 3.3. Characterization of MCNs

SEM images were obtained using a field emission scanning electron microscope (ULTRA55, ZEISS) operated at 3.0 kV. TEM images were captured using a transmission electron microscope (JEM2100, TOSHIBA). The specific surface area and pore size distribution were determined by Quadrasorb evo^TM^ (Quantachrome). Before the adsorption, the samples were degassed at 120 °C for 8 h. The specific surface areas were calculated by the multipoint Brunauer–Emmett–Teller (BET) method and the pore size distribution was calculated by the discrete Fourier transform (DFT) method. The total pore volume was estimated from the N_2_ amount adsorbed at a relative pressure of P/P_0_ = 0.99. The small-angle X-ray scattering (SAXS) measurements used 13.5 keV radiation (λ = 0.918 Å). Raman spectra measurements were performed using the RenishawinVia + Reflex Raman spectrometer at a 514 nm excitation wavelength with a frequency range of 1000–2000 cm^−1^. The infrared spectrum was obtained by the Thermo Scientific Nicolect IS10 Fourier transform infrared spectrometer. The X-ray diffraction (XRD) measurements were taken with a Rigaku D/max B diffractometer using Cu KR radiation (40 kV, 20 mA). 

### 3.4. Adsorption Properties for Antibiotics Solution

The adsorption study of antibiotics was carried out using 0.01 g of MCNs loaded in 100 mL with the concentrations of streptomycin, penicillin, and tetracycline hydrochloride ranging from 25 to 600 mg/L. The mixture was continuously stirred in a Thermostatic Magnetic Mixer (DF-101S, Shanghai Yuezhong Instrument and Equipment Co., Ltd., Shanghai, China) with a speed of 120 rpm/min for 12 h. After adsorption, the concentration of the antibiotic solution was measured under normal incident light by the Lambda750 infrared–visible–ultraviolet spectrometer at 266 nm, 282 nm, and 365 nm, respectively. The limits of quantification (LOQs) of penicillin, streptomycin, and tetracycline hydrochloride were about 40.0 mg/L, 5.0 mg/L, and 30.0 mg/L, respectively. The adsorption capacity at equilibrium time *Q_e_* (mg/g) and any time *Q_t_* (mg/g) and the removal efficiency (%) were calculated according to the following equations:

(9) $$Q_{e}=\frac{(C_{0}-C_{e})V}{m}$$

(10) $$Q_{t}=\frac{(C_{0}-C_{t})V}{m}$$

(11) $$\mathrm{Removal}\;\mathrm{efficiency}\;\left(\%\right)=\frac{\left(C_{0}-C_{e}\right)}{C_{0}}\times 100$$

where C_0_ (mg/L), C_e_ (mg/L), and C_t_ (mg/L) are the initial concentration, the equilibrium concentration, and the concentration of antibiotics in water, respectively. *V* (L) is the volume of the antibiotic solutions, and m (g) is the weight of the adsorbent. The effect of contact time on adsorption was also studied, and the adsorption amount was analyzed according to Equation (10).

The recycling test for antibiotic adsorption was carried out in three parallel experiments. The antibiotic-loaded MCNs were dispersed in ethanol for 12 h and then washed three times with ethanol and distilled water. After that, the adsorbents were dried in a vacuum oven at 60 °C.

## 4. Conclusions

We developed high-purity MCNs for the removal of antibiotics from water environments. High-purity MCNs were synthesized by exploiting F127 and phenolic resin. MCNs with uniform pores were obtained based on the combination of the self-assembling approach and the hydrothermal method. The prepared MCNs have a high surface area and a large pore volume. They also exhibit high adsorption capacities and high removal efficiency in adsorbing antibiotics. The adsorption capacity of tetracycline hydrochloride can reach up to 880.6 mg/g. The high adsorption capacity and removal efficiency are attributed not only to rich annular mesoporosity, high surface area, and large pore volumes, but also high-purity MCNs have more absorption sites. Notably, the synthesized MCNs have a higher adsorption rate and adsorption capacity for tetracycline hydrochloride due to their rich annular mesoporosity and special mesoporous diameter. Furthermore, the MCNs can be simply regenerated and recovered. After five adsorption and regeneration cycles, the adsorption capacity could still reach 76.6% with stable performance. The high removal efficiency of penicillin, streptomycin, and tetracycline hydrochloride residues indicates that the prepared MCNs have potential as promising adsorbents in water environment applications.

## Figures and Tables

**Table 1:** The parameters of Langmuir and Freundlich isotherm of three antibiotics’ adsorption on MCNs.

Name	Langmuir Isotherm			Freundlich Isotherm		
	*k_L_* (L/mg)	Q_m__,_ (mg/g)	R^2^	n	*k_f_* (mg/g)/(mg/ L)^n^	R^2^
Tetracycline hydrochloride	6.75 × 10^−6^	31,497	0.994	1.07	0.349	0.994
Penicillin	1.88 × 10^−4^	693	0.945	1.49	1.121	0.957
Streptomycin	1.17 × 10^−6^	8483	0.819	1.17	0.034	0.823

**Table 2:** The adsorption kinetic parameters of three antibiotics on MCNs.

Name	Pseudo-First-Order				Pseudo-Second-Order		
	Q_e__,__exp_ (mg/g)	Q_e__,__cal_ (mg/g)	*k_1_* (h^−1^)	R^2^	Q_e_ (mg/g)	*k_2_* (h^−1^)	R^2^
Tetracycline hydrochloride	4268.2	3744.3	1.51	0.903	1480.9	5.83 × 10^−5^	0.013
Penicillin	886.3	1010.3	0.99	0.830	2283.1	1.54 × 10^−5^	0.037
Streptomycin	256.2	123.5	0.71	0.840	71,994.2	1.86 × 10^−9^	0.020

**Table 3:** The intra-particle diffusion model parameters of three antibiotics on MCNs.

Name	Intra-Particle Diffusion								
	*k_1_* (mg/g h^−1/2^)	c (mg/g)	R^2^	*k_2_* (mg/g h^−1/2^)	c (mg/g)	R^2^	*k_3_* (mg/g h^−1/2^)	c (mg/g)	R^2^
Tetracycline hydrochloride	721.50	−499.85	0.96	577.71	−292.22	0.96	4.86	868.54	0.89
Penicillin	134.84	−58.40	0.98	339.65	−340.84	0.99	47.24	236.15	0.88
Streptomycin	9.77	−0.28	0.98	59.18	71.29	0.96	17.01	14.12	0.97

## Data Availability

The data used to support the findings of this study are available on request from the corresponding author.

## Sample Availability

Not available.

## References

[1] Hutchings M.I., Truman A.W., Wilkinson B. (2019). Antibiotics: Past, present and future. Curr. Opin. Microbiol..

[2] Huang A., Yan M., Lin J., Xu L., Gong H., Gong H. (2021). A review of processes for removing antibiotics from breeding wastewater. Int. J. Environ. Res. Public Health.

[3] Phoon B.L., Ong C.C., Mohamed Saheed M.S., Show P.-L., Chang J.-S., Ling T.C., Lam S.S., Juan J.C. (2020). Conventional and emerging technologies for removal of antibiotics from wastewater. J. Hazard. Mater..

[4] Kovalakova P., Cizmas L., McDonald T.J., Marsalek B., Feng M., Sharma V.K. (2020). Occurrence and toxicity of antibiotics in the aquatic environment: A review. Chemosphere.

[5] Lyu J., Yang L., Zhang L., Ye B., Wang L. (2020). Antibiotics in soil and water in China—A systematic review and source analysis. Environ. Pollut..

[6] Li S., Show P.L., Ngo H.H., Ho S.H. (2022). Algae-mediated antibiotic wastewater treatment: A critical review. Environ. Sci. Ecotechnol..

[7] Chu L., Wang J., He S., Chen C., Wojnárovits L., Takács E. (2021). Treatment of pharmaceutical wastewater by ionizing radiation: Removal of antibiotics, antimicrobial resistance genes and antimicrobial activity. J. Hazard. Mater..

[8] Wang J., Zhuan R. (2020). Degradation of antibiotics by advanced oxidation processes: An overview. Sci. Total Environ..

[9] Mahdi M.H., Mohammed T.J., Al-Najar J.A. (2021). Advanced oxidation processes (AOPs) for treatment of antibiotics in wastewater: A review. IOP Conf. Ser. Earth Environ. Sci..

[10] Mangla D., Annu Sharma A., Ikram S. (2022). Critical review on adsorptive removal of antibiotics: Present situation, challenges and future perspective. J. Hazard. Mater..

[11] Torkian N., Bahrami A., Hosseini-Abari A., Momeni M.M., Abdolkarimi-Mahabadi M., Bayat A., Hajipour P., Amini Rourani H., Abbasi M.S., Torkian S. (2022). Synthesis and characterization of Ag-ion-exchanged zeolite/TiO2 nanocomposites for antibacterial applications and photocatalytic degradation of antibiotics. Environ. Res..

[12] Manjunath S.V., Singh Baghel R., Kumar M. (2020). Antagonistic and synergistic analysis of antibiotic adsorption on Prosopis juliflora activated carbon in multicomponent systems. Chem. Eng. J..

[13] Ma J., Jiang Z., Cao J., Yu F. (2020). Enhanced adsorption for the removal of antibiotics by carbon nanotubes/graphene oxide/sodium alginate triple-network nanocomposite hydrogels in aqueous solutions. Chemosphere.

[14] Krasucka P., Pan B., Ok Y.S., Mohan D., Sarkar B., Oleszczuk P. (2021). Engineered biochar—A sustainable solution for the removal of antibiotics from water. Chem. Eng. J..

[15] Wang H., Li Z., Yahyaoui S., Hanafy H., Seliem M.K., Bonilla-Petriciolet A., Luiz Dotto G., Sellaoui L., Li Q. (2021). Effective adsorption of dyes on an activated carbon prepared from carboxymethyl cellulose: Experiments, characterization and advanced modelling. Chem. Eng. J..

[16] Jawad A.H., Saud Abdulhameed A., Wilson L.D., Syed-Hassan S.S.A., Alothman Z.A., Rizwan Khan M. (2021). High surface area and mesoporous activated carbon from KOH-activated dragon fruit peels for methylene blue dye adsorption: Optimization and mechanism study. Chin. J. Chem. Eng..

[17] Wang B., Xu X., Tang H., Mao Y., Chen H., Ji F. (2020). Highly efficient adsorption of three antibiotics from aqueous solutions using glucose-based mesoporous carbon. Appl. Surf. Sci..

[18] Xu L., Zhang M., Wang Y., Wei F. (2021). Highly effective adsorption of antibiotics from water by hierarchically porous carbon: Effect of nanoporous geometry. Environ. Pollut..

[19] Fang Y., Gu D., Zou Y., Wu Z., Li F., Che R., Deng Y., Tu B., Zhao D. (2010). A low-concentration hydrothermal synthesis of biocompatible ordered mesoporous carbon nanospheres with tunable and uniform size. Angew. Chem. Int. Ed..

[20] Liu J., Xie L., Deng J., Gong Y., Tang G., Bai H., Wang Y. (2019). Annular mesoporous carbonaceous nanospheres from biomass-derived building units with enhanced biological interactions. Chem. Mater..

[21] Yang X., Lu P., Yu L., Pan P., Elzatahry A.A., Alghamdi A., Luo W., Cheng X., Deng Y. (2020). An Efficient emulsion-induced interface assembly approach for rational synthesis of mesoporous carbon spheres with versatile architectures. Adv. Funct. Mater..

[22] Ferrari A.C., Robertson J. (2000). Interpretation of Raman spectra of disordered and amorphous carbon. Phys. Rev. B.

[23] Wen Y.R., Liu Y., Liu D.H., Tang B., Liu C.T. (2011). Microstructural evolution of ferritic steel powder during mechanical alloying with iron oxide. Int. J. Mater. Res..

[24] An K., Xu X. (2019). Mo_2_C based electrocatalyst with nitrogen doped three dimensional mesoporous carbon as matrix, synthesis and HER activity study. Electrochim. Acta.

[25] Yang J., Dou Y., Yang H., Wang D. (2021). A novel porous carbon derived from CO 2 for high-efficient tetracycline adsorption: Behavior and mechanism. Appl. Surf. Sci..

[26] Cheng D., Ngo H.H., Guo W., Chang S.W., Nguyen D., Zhang X., Varjani S., Liu Y. (2020). Feasibility study on a new pomelo peel derived biochar for tetracycline antibiotics removal in swine wastewater. Total Environ..

